# Evaluation of Fracture Resistance in Teeth with Iatrogenic Perforations Repaired Using the Newly Developed Materials NeoPUTTY and NeoMTA 2: An In Vitro Comparative Study

**DOI:** 10.3390/dj14070437

**Published:** 2026-07-14

**Authors:** Flora Kakoura, Kleoniki Lyroudia, Nikolaos Economides, Georgios Mikrogeorgis

**Affiliations:** Department of Endodontology, School of Dentistry, Aristotle University of Thessaloniki, 54124 Thessaloniki, Greece; fkakour@dent.auth.gr (F.K.); econom@dent.auth.gr (N.E.)

**Keywords:** NeoMTA 2, NeoPUTTY, Biodentine, fracture resistance, root perforation, calcium silicate materials

## Abstract

**Objectives:** This study aimed to assess and compare the fracture resistance (FR) of teeth with simulated root perforations repaired using NeoMTA 2, NeoPUTTY, and Biodentine. **Methods:** Sixty-five single-rooted human teeth were decoronated to a standardized length of 13 mm from the apex. Their weight, mesiodistal (MD) and buccolingual (BL) dimensions were recorded for even distribution across five groups (n = 13). The teeth in the three experimental groups were instrumented. Standardized 1 mm perforations were created on the buccal root surface at a fixed 105° angle using a precision apparatus. The canals were then obturated with gutta-percha and AH Plus sealer. The perforations were subsequently repaired with NeoMTA 2, NeoPUTTY, or Biodentine. The positive control (PC) group included instrumented and perforated roots, while the negative control (NC) group comprised untreated, intact roots. All control group data used in this study were derived from previously published research. Fracture testing was conducted with a universal testing machine applying a compressive vertical load at 1 mm/min until fracture occurred. The forces were analyzed using one-way analysis of variance (ANOVA), followed by a post hoc Tukey test. **Results:** The mean fracture loads were as follows: NC Group: 342.68 ± 146.45 N, PC Group: 212.66 ± 77.89 N, NeoMTA 2 Group: 314.34 ± 131.41 N, NeoPUTTY Group: 243.76 ± 93.71 N, and Biodentine Group: 263.73 ± 94.63 N. No statistically significant differences were observed among experimental groups (*p* > 0.05). **Conclusions:** The three biomaterials showed similar strengthening effects but did not fully restore fracture strength.

## 1. Introduction

Root perforation during endodontic treatment is a procedural complication that creates an artificial communication between the root canal system and the periodontium. The success of its management depends on the size and location of the perforation and requires immediate hermetic sealing [1]. Mineral trioxide aggregate (MTA) has long been the material of choice for sealing root perforations due to its excellent biocompatibility, sealing capacity, and pronounced bioactivity [2]. However, despite its numerous advantages, MTA presents several limitations, including difficult handling, potential tooth discoloration, a prolonged setting time [3], and possible disintegration when exposed to tissue fluids [4]. To address these limitations, other calcium silicate-based materials have been developed. Biodentine (Septodont, Saint Maur-des-Fosses, France) is a high-purity bioceramic cement, available as a powder-liquid system, proposed as a dentin substitute for both crown and root applications. Compared to MTA, Biodentine demonstrates a shorter setting time and improved handling characteristics [5]. In addition, its compressive strength (304 MPa) and elastic modulus (22 GPa) closely approximate those of human dentin (297 ± 24 MPa and 14–18.6 GPa, respectively) [5,6].

To meet the demand for bioactive materials with improved physical properties, two novel bioceramic products have recently become commercially available. NeoMTA 2 (NuSmile, Houston, TX, USA) is a powder–gel system cement primarily composed of ultrafine tricalcium and dicalcium silicate particles. In addition, tantalite is used as a radiopacifying agent, replacing the bismuth oxide found in conventional MTA formulations, which is associated with tooth discoloration [7,8]. NeoPUTTY (NuSmile, Houston, TX, USA) is a low-tack putty available in a pre-mixed, syringeable format, with a composition closely resembling that of NeoMTA 2 [9]. The cytocompatibility of these novel materials is adequate and comparable to that of their predecessor, NeoMTA Plus [10,11]. According to the manufacturer, both products are immediately washout-resistant, dimensionally stable and exhibit low water solubility (<3%) upon setting [7,9]. However, these manufacturer-reported properties warrant independent validation.

Teeth with extensive root defects, such as resorptive lesions, exhibit reduced fracture resistance (FR) and are more susceptible to structural failure [2,12,13]. Similarly, an iatrogenic root perforation may complicate the prognosis of endodontic therapy—not only due to potential periodontal involvement, but also because it compromises the mechanical integrity of the affected tooth. The restorative potential of the tooth may be enhanced by the material used to seal the perforation. Ideally, the repair material should reinforce the weakened root and improve its resistance to vertical fracture [12].

Studies evaluating the reinforcing potential of Biodentine in structurally compromised roots have been limited to teeth with internal resorption cavities [2,12,13]. Owing to their recent introduction, scientific data on the physical properties of the newly developed NeoMTA 2 and NeoPUTTY remain limited [10,11,14,15]. Although all the aforementioned biomaterials share calcium silicate-based compositions, they differ chemically in several aspects (Table 1). It has been suggested that variations in particle size or powder-to-liquid ratios may influence a cement’s compressive strength [8]. Consequently, it remains unclear whether these compositional differences affect the mechanical properties and reinforcing potential of the materials. To the best of our knowledge, this is the first study to evaluate and compare the reinforcing effect of the recently introduced calcium silicate-based materials NeoMTA 2 and NeoPUTTY with that of Biodentine on the FR of roots with simulated iatrogenic perforations. The null hypothesis was that the three biomaterials would not differ significantly in their effect on FR.

## 2. Materials and Methods

### 2.1. Sample Selection and Preparation

The present investigation adhered to the principles of the Declaration of Helsinki and was approved by the Ethics Committee of the Dental School of Aristotle University of Thessaloniki (protocol no. 126/14-07-2021). All procedures were conducted at the School of Dentistry, Faculty of Health Sciences, Aristotle University of Thessaloniki, Greece.

A previous companion study by the same research team, published in *Dentistry Journal*, demonstrated that teeth with 1 mm iatrogenic perforations exhibited a statistically significant reduction in FR compared to intact teeth. The specimens and findings from that study were incorporated into the present study design. Specifically, intact teeth are employed as the negative control (NC) group, while instrumented teeth with iatrogenic perforations serve as the positive control (PC) group [16]. It should be noted that all specimens used across both the present and the companion study were derived from a standardized experimental pool and processed under identical conditions. More specifically, all specimens were prepared, stored, and subjected to mechanical testing during the same experimental period by the same operators, using the same equipment, under identical environmental conditions, and following an identical experimental protocol. This approach minimizes redundancy, enhances methodological consistency, and reduces the number of human tooth specimens required, in accordance with ethical principles for the responsible use of human biological material.

The required sample size was calculated based on pilot study data using G*Power 3.1 software (ANOVA, F-tests; effect size = 0.572; power (1 − β) = 0.95; α = 0.05), resulting in a total of 65 specimens.

Sixty-five single-rooted teeth extracted due to periodontal disease were selected for the study. Only teeth meeting the inclusion criteria of structural integrity and absence of caries, cracks, or internal or external resorption were selected. Following extraction, the teeth were cleaned and preserved in a 0.1% thymol solution until use and for a maximum duration of two months [17]. Buccolingual (BL) and mesiodistal (MD) radiographs for each sample verified the existence of single root canals without pathologic resorption or calcification. The surface of the samples was inspected at ×10 magnification for cracks and flaws with the aid of a stereomicroscope (Stemi 2000C, Carl Zeiss, Oberkochen, Germany). All teeth were sectioned at 13 mm from the apex using a diamond sectioning blade (Isomet, Buehler, Waukegan, IL, USA) under continuous water cooling.

The roots were then randomly assigned to five equal groups (n = 13) using a computer-generated randomization sequence. For each specimen, the weight, BL and MD dimensions were measured using a precision analytical scale and an electronic digital caliper, respectively. These measurements were subjected to statistical analysis to verify dimensional homogeneity both within and between groups, as detailed in Section 2.4.

Each tooth was wrapped in a single sheet of lead and positioned perpendicularly into a rubber mold filled with cold self-curing acrylic resin (BMS 015 powder/liquid; BMS Dental, Capannoli, Italy), leaving approximately 1 mm of the coronal third exposed. After polymerization, the lead sheet was removed, and radiographic imaging was performed using the parallel cone technique at BL and MD angulations to confirm the perpendicular alignment of the teeth within the acrylic blocks [18].

The NC group consisted of 13 teeth that remained intact [16]. In the remaining specimens, the working length was negotiated with a #10-K stainless steel file (Dentsply, Maillefer, Ballaigues, Switzerland), positioned 1 mm short of the apical foramen. Root canal instrumentation was carried out using rotary files (ESX^®^ System; Brasseler USA, Savannah, GA, USA) up to size #35/0.04. Mechanical shaping was accompanied by continuous irrigation with 3 mL of 2.5% sodium hypochlorite. Following instrumentation, all roots were subjected once more to stereomicroscopic evaluation for the detection of newly developed cracks [19].

### 2.2. Perforation Simulation and Root Sealing

**Figure 1:**
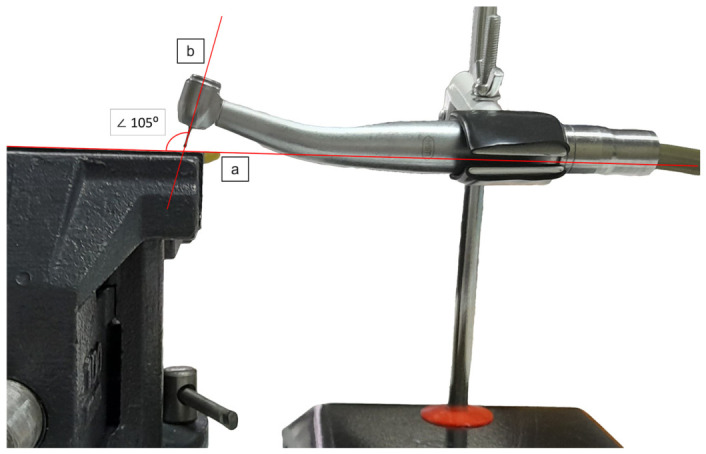
Part of the experimental setup illustrating the spatial relationship between the buccal surface of the root (**a**) and the axis of the cutting bur (**b**). To enhance visual clarity, the image background was digitally removed using Adobe Photoshop CC (Adobe Systems Inc., San Jose, CA, USA).

To ensure consistent perforations, the high-speed handpiece was aligned horizontally and secured in a custom-made apparatus that permitted only vertical downward movement. The teeth were stabilized in a vise with their buccal surfaces oriented parallel to the ground. This setup created a fixed angle of 105° between the buccal surface of the specimens and the shank of the cutting bur (Figure 1). Diagonal 1 mm perforations were created using water-cooled round burs (G801010, JTC Fulldent SA, Arzier, Switzerland), initiated approximately 3 mm apical to the coronal surface of the roots. Consequently, all simulated defects were uniform in both direction and diameter. To prevent overextension of the perforation into the lingual dentin, a #35-K hand file (Dentsply, Maillefer, Ballaigues, Switzerland) was temporarily placed in the root canal. Each bur was used for a single preparation and then discarded. Thirteen teeth subjected to chemomechanical root canal preparation and a standardized 1 mm perforation, without repair, served as the PC group [16].

The remaining root canals were dried with paper points and obturated using the single-cone technique with #35/0.04 gutta-percha master cones (Cerkamed, Stalowa Wola, Poland) and AH Plus sealer (Dentsply DeTrey, Konstanz, Germany). The gutta-percha cones were cut 1 mm apical to the canal orifice using the System B Heat Source (SybronEndo, Glendora, CA, USA). During obturation, strips of Teflon tape were temporarily compacted into the perforation sites to prevent sealer intrusion. Prior to sealing the perforations, the teeth were wrapped in moistened gauze and stored in an incubator at 37 °C and 100% humidity for 48 h to allow complete setting of the sealer.

**Figure 2:**
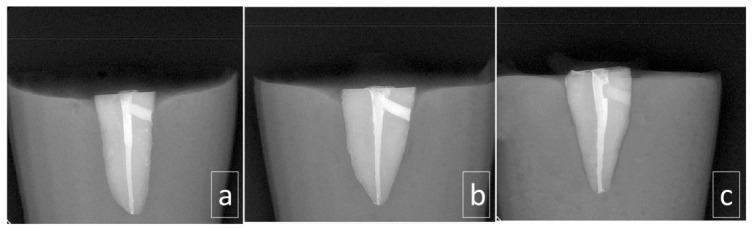
Representative specimens from the experimental groups: (**a**) NeoMTA 2, (**b**) NeoPUTTY, and (**c**) Biodentine. Note the differences in radiopacity among the three bioceramic materials.

The three experimental groups were established based on the biomaterial used to seal the perforations: the NeoMTA 2 Group, the NeoPUTTY Group, and the Biodentine Group (Figure 2). The bioceramic materials were prepared in accordance with the manufacturers’ instructions (Table 1) and incrementally placed into the defects using hand pluggers. Subsequently, the coronal access cavities were sealed with Cavit G (3M ESPE, St. Paul, MN, USA). The specimens were then wrapped in saline-soaked gauze and stored again in the incubator for one month until the FR test.

### 2.3. Fracture Resistance Test

**Figure 3:**
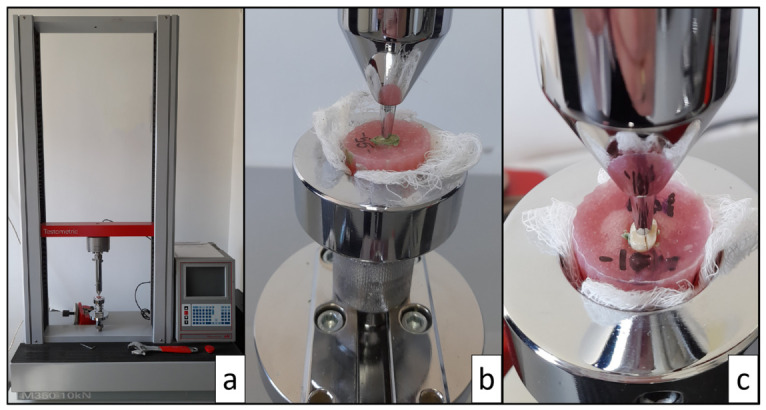
Representative images of the experimental setup: (**a**) a specimen mounted on the universal testing machine; (**b**) the fracture test probe with the 0.5 mm diameter conical tip accurately aligned over the root canal orifice; (**c**) buccolingual vertical root fracture observed following compressive load application.

To mimic the periodontal ligament, a thin layer of vinyl polysiloxane impression material (3M Express Light Body, 3M ESPE, Saint Paul, MN, USA) was applied to the root surfaces and used to fill the vacant space between the teeth and their artificial sockets [20]. The specimens were mounted on the lower platen of a universal testing machine (Testometric M350-10 KN, Linkon Close, Rochdale, UK). A stainless-steel cylindrical probe, with a 60° conical tip (0.5 mm tip diameter), was centrally aligned over the root canal orifice. A static vertical compressive load was applied at a crosshead speed of 1 mm/min until root fracture occurred (Figure 3). The maximum force required to induce fracture was recorded in newtons (N).

### 2.4. Statistical Analysis

At the outset of the experiment, both intragroup and intergroup dimensional homogeneity of the specimens was assessed. Specifically, box plot analysis was conducted to identify potential outliers within the groups. Furthermore, the Shapiro–Wilk test verified the normal distribution of all the involved variables (*p* > 0.05). Accordingly, parametric one-way analysis of variance (ANOVA) was applied to detect statistically significant differences among groups.

Regarding the force-to-fracture values (N), box plot analysis was used to assess the presence of outliers within each group. Normality of the data was confirmed using the Shapiro–Wilk test (*p* > 0.05). One-way ANOVA was performed to compare the mean values among the groups, followed by Tukey’s HSD post hoc test for multiple pairwise comparisons. Statistical analyses were conducted using IBM SPSS Statistics software (Version 25), with the significance level set at 5%.

## 3. Results

Dimensional homogeneity was confirmed, as no intragroup outliers or statistically significant intergroup differences were detected (*p* > 0.05). Regarding FR values, the ANOVA test revealed a statistically significant difference among groups (*p* < 0.05). According to Tukey’s HSD test, the NC group exhibited a significantly higher mean FR value than the PC group (*p* < 0.05). Among the experimental groups, the NeoMTA 2 group showed the highest mean FR value, followed by the Biodentine and NeoPUTTY groups, respectively. However, no statistically significant differences were found among the three experimental groups (*p* > 0.05). These results suggest that all three biomaterials provided comparable reinforcement; however, none fully restored FR. The descriptive statistics and the ANOVA results for the variables examined in the study are presented in Table 2.

## 4. Discussion

Effective management of root perforations requires the application of a bioactive repair material that not only ensures a reliable seal but also reinforces the residual tooth structure to prevent fracture. Although the reinforcing properties of Biodentine have been explored, the current literature is confined to roots affected by pathologic resorptive defects [2,12,13,21]. Due to their recent commercialization, studies assessing the physical behavior of the novel tantalite-containing bioceramic materials NeoMTA 2 and NeoPUTTY remain limited [11,14,15,22]. Although these materials share a calcium silicate-based composition, they incorporate distinct additives that may influence their physical performance, including compressive strength [8]. Accordingly, this in vitro study was designed to assess the reinforcing effect of Biodentine, NeoMTA 2, and NeoPUTTY on the FR of roots with simulated iatrogenic perforations.

It has been stated that adequate adhesion of a material to the root canal dentin increases its reinforcing ability [12]. As observed in the current study, the experimental groups exhibited higher FR values compared to the PC group, although the difference was not statistically significant. Gutta-percha, lacking the ability to bond to dentin, has been shown to provide minimal reinforcement following root canal therapy [23]. Consequently, the improved performance observed in the experimental groups may be attributed to the chemical bonding between calcium silicate-based cements and the dentinal walls [24]. This interpretation is further supported by a finite element analysis study, which revealed that sealing root resorptions with MTA, rather than gutta-percha, resulted in more uniform stress distribution and a notable reduction in stress concentrations within the remaining root structure [25].

Previous studies have shown that Biodentine demonstrated higher FR values compared to RetroMTA and Portland cement [13], as well as MTA and MTA Plus [2], with no statistically significant differences among the materials in either case. The comparable FR values among calcium silicate-based cements align with the findings of our study, where the NeoMTA 2 group showed a higher mean FR value than Biodentine and NeoPUTTY, though the difference was not considered significant. Based on the present findings, the null hypothesis could not be rejected, as no statistically significant differences were observed among the three tested biomaterials.

NeoMTA Plus, the predecessor of NeoMTA 2, achieved greater penetration into the dentinal tubules than Biodentine; however, the observed difference lacked statistical relevance [26]. As claimed by the manufacturer, NeoMTA 2 is composed of extremely fine calcium-silicate powder [7]. Hence, its penetration into dentin could result in increased surface contact. Moreover, a study reported that NeoMTA 2 demonstrated enhanced mineralization potential compared to NeoMTA Plus, which may be attributed to its increased tantalite content and the inclusion of distinct polymers in its formulation [11]. Consequently, the release of large amounts of calcium ions from NeoMTA 2 [11], along with the presence of small particle sizes that increase the specific surface area of the material [27], might enhance water adsorption and hydration reactions, potentially promoting the formation of the interfacial layer and improving bonding with dentin. The elevated FR values observed in the NeoMTA 2 group could be associated with these material characteristics.

The manufacturer of NeoPUTTY claims that it has several advantages over NeoMTA 2, as it is 25% more radiopaque (Figure 2) and does not require mixing before use [9]. Despite these claimed benefits, our study revealed that NeoPUTTY exhibited a lower mean FR value compared to NeoMTA 2; although the difference was not statistically significant. The setting reaction of NeoMTA 2 is initiated immediately upon mixing the powder with the water-based gel and continues in vivo in the presence of moisture from the dentinal tubules, periodontium, or pulp. In contrast, NeoPUTTY is formulated with a non-aqueous organic liquid and sets solely in response to moisture from the surrounding environment. In a recent study, while EndoSequence BC RRM Putty fully hardened within 10 days of exposure to ambient air, NeoPUTTY exhibited only partial setting even after 30 days, supporting the manufacturer’s claim of a longer shelf life [14]. In our study, the hydration reaction of the experimental materials occurred in the presence of a gauze soaked in phosphate-buffered saline, which was wrapped around the external surface of the root. Since the materials were placed in diagonal tunnel root defects, it remains unclear whether the limited contact of synthetic tissue fluid with only the outermost portion of NeoPUTTY was sufficient to induce complete setting. Evidence suggests that insufficient hydration during setting may reduce the ultimate strength of the material [13], which may have contributed to the lower mean FR values observed in this study.

This investigation was carried out with increased efforts to standardize the parameters of the FR test, which is essential when evaluating the load-bearing capacity of natural teeth [28]. Given the considerable variation in tooth dimensions among individuals, significant differences in root dimensions between study groups may influence the force values recorded during the experiment. In our study, the coronal BL and MD diameters, along with root weight, were measured and, importantly, statistically analyzed to ensure dimensional homogeneity among groups. Additionally, the teeth were initially stored in a 0.1% thymol solution for up to eight weeks, as this medium and storage duration have been shown not to significantly affect dentin microhardness [17].

A custom-made apparatus was employed to standardize the creation of simulated iatrogenic perforations. A high-speed handpiece was securely fixed in a horizontal position, permitting only vertical downward movement. This setup ensured a consistent angle between the buccal surface of the roots and the shank of the cutting bur during drilling. As a result, all perforations were created with uniform geometry and straight outlines, effectively minimizing operator bias and simulating the clinical presentation of diagonal root perforations. If the cavities were manually created by an operator holding the dental drilling device directly, this approach might have introduced variability in angulation, depth, and position, potentially compromising standardization and reducing the reproducibility of the results. In previous studies, details regarding the preparation of radicular cavities are limited [2,13].

Another notable distinction in this experiment is that the teeth were stored in an incubator for 30 days prior to FR testing to allow for the complete setting of the materials. This time interval was selected based on scientific evidence indicating that the compressive strength of Biodentine increases over time, reaching approximately 300 MPa after one month [5]. Although comparable data regarding the compressive strength of NeoMTA 2 and NeoPUTTY are not available in the literature, the same storage period was applied to all materials to maintain standardization. In relevant FR studies, the incubation period of Biodentine specimens prior to load application has varied, ranging from 48 h [12] to 7 days [2,13] and up to 14 days [21].

The simulation of the alveolar process and periodontal ligament is recommended during static load testing [20]. Accordingly, a polymethylmethacrylate acrylic resin was used to replicate the bone socket, while a vinyl polysiloxane impression material was employed to simulate the periodontal tissue. Moreover, the design of the load application probe allowed precise alignment over the root canal orifices and ensured the application of vertical forces. Although oblique loading more closely reflects clinical conditions, the consistent vertical loading angle contributed to the standardization of the monotonic test. It has been stated that the probe design used in monotonic tests can impact the resulting FR measurements [29].

One limitation of this in vitro study is the limited comparability of its findings, given the scarcity of studies evaluating the reinforcing ability of novel materials NeoMTA 2 and NeoPUTTY in teeth. It should also be recognized that laboratory studies are inherently limited in their ability to replicate the full complexity of clinical conditions; consequently, the results may not directly reflect in vivo performance. Furthermore, the present study employed a static vertical loading protocol, whereas occlusal forces in the oral environment are predominantly cyclic and multidirectional. Consequently, the reinforcing behavior of the tested materials under fatigue loading may differ from that observed under static monotonic loading conditions. Despite these limitations, in vitro studies remain valuable, as they offer controlled conditions for isolating specific variables—such as perforation repair materials—and evaluating their effect on the structural behavior of endodontically treated teeth. Accordingly, the results of this study provide a basis for further in vivo and clinical investigations to confirm their clinical relevance. Taken together, the present findings contribute to the growing body of evidence on the mechanical behavior of calcium silicate-based repair materials and enhance the current understanding of their behavior in structurally compromised teeth.

## 5. Conclusions

Within the limitations of this methodology, all tested calcium silicate-based materials demonstrated comparable effectiveness in reinforcing roots with simulated iatrogenic perforations, with no statistically significant differences observed in FR. Importantly, none of the materials was able to fully restore the original FR of the tooth. Consequently, all tested materials may be considered suitable alternatives for the repair of root perforations, exhibiting similar mechanical behavior under the conditions of this study.

## Figures and Tables

**Table 1:** Chemical composition and mixing procedure of the tested materials.

Bioceramic	Composition	Mixing
**Biodentine** [5]	**Powder:** Tricalcium silicate, Dicalcium silicate, Zirconium oxide, Calcium carbonate, Calcium oxide, Iron oxide **Liquid:** Calcium chloride, Polycarboxylate	**Capsule product**. 5 drops of liquid: 1 capsule with premeasured powder. The capsule is triturated for 30 s on a mixing device at a speed of 4000 rotations/min.
**NeoMTA 2** [7]	**Powder**: Tricalcium silicate, Dicalcium silicate, Tantalite, Calcium sulfate, Tricalcium aluminate **Gel:** Water and proprietary ingredients	**Hand-mixed product**. 1 scoop (0.05–0.1 g) of powder: 1 or 2 drops of gel. Gradual incorporation of gel into powder; variable powder/gel ratio.
**NeoPUTTY** [9]	**Putty:** Tricalcium silicate, Dicalcium silicate, Tantalite, Calcium sulfate, Tricalcium aluminate, Calcium aluminate, Grossite	**Pre-mixed product**. Ready to use.

**Table 2:** Mean and standard deviation values of the buccolingual (BL) and mesiodistal (MD) dimensions, the weight, and the force-to-fracture of the study groups.

Groups	Negative Control [16]	Positive Control [16]	NeoMTA 2	NeoPUTTY	Biodentine	ANOVA (*p*-Values)
**BL (mm)**	7.82 ± 0.55	7.70 ± 0.56	7.73 ± 0.48	7.70 ± 0.70	7.63 ± 0.44	0.937
**MD (mm)**	4.62 ± 0.15	4.72 ± 0.29	4.62 ± 0.28	4.66 ± 0.43	4.41 ± 0.35	0.159
**Weight (g)**	0.45 ± 0.02	0.44 ± 0.05	0.44 ± 0.04	0.44 ± 0.04	0.45 ± 0.05	0.952
**Force (N)**	342.68 ± 146.45 ^a^	212.66 ± 77.89 ^b^	314.34 ± 131.41 ^ab^	243.76 ± 93.71 ^ab^	263.73 ± 94.63 ^ab^	0.029 *

**Note**: mm = millimeter; g = gram; N = newtons. * Significant at 0.05 level. Different superscript letters indicate statistically significant differences between groups.

## Data Availability

The datasets generated and analyzed during the current study are not publicly available due to their inclusion in a Ph.D. project in progress, but they are available from the corresponding author upon reasonable request.

## Abbreviations

The following abbreviations are used in this manuscript:

FR	Fracture resistance
MD	Mesiodistal
BL	Buccolingual
PC	Positive control
NC	Negative control
ANOVA	Analysis of variance
MTA	Mineral trioxide aggregate
N	Newtons
mm	Millimeter
g	Gram
